# FLT3LG modulates the infiltration of immune cells and enhances the efficacy of anti-PD-1 therapy in lung adenocarcinoma

**DOI:** 10.1186/s12885-025-14220-x

**Published:** 2025-05-06

**Authors:** Fengyu Zhao, Han Bai, Yiwei Liu, Shuoze Gao, Chengcheng Yang, Jie Wu, Hao Cheng, Jiao Ma, Yuanyuan Li, Hong Ren, Junke Fu, Shanzhi Gu, Xinhan Zhao, Sida Qin

**Affiliations:** 1 Department of Oncology, The First Affiliated Hospital of Xi’an Jiaotong University, Xi’an, Shaanxi Province 710061 China; 2 The MED-X Institute, The First Affiliated Hospital of Xi’an Jiaotong University, Building 21, Xi’an, China; 3 Department of Thoracic Surgery, The First Affiliated Hospital of Xi’an Jiaotong University, 277 West Yanta Road, Xi’an, Shaanxi Province 710061 China; 4 Department of Radiation Oncology, Shaanxi Provincial People’s Hospital, Xi’an, Shaanxi Province China; 5 Department of Rehabilitation, The First Affiliated Hospital of Xi’an Jiaotong University, Xi’an, Shaanxi Province 710061 China; 6 Department of Forensic Medicine, Medical School of Xi’an Jiaotong University, Xi’an, Shaanxi Province 710061 China

**Keywords:** Cancer immunotherapy, FLT3LG, Lung adenocarcinoma (LUAD), Biomarkers, Immune cell infiltration

## Abstract

**Background:**

Immunotherapy, particularly anti-PD-1 therapy, has assumed a progressively significant position in the management of non-small cell lung cancer (NSCLC), especially in lung adenocarcinoma (LUAD). Nevertheless, a subset of patients exhibit resistance to anti-PD-1 therapy, and the exploration of biomarkers for evaluating the responsiveness to anti-PD-1 therapy necessitates further investigation. *FLT3LG* is regarded as being associated with tumor diagnosis and immunotherapy in a variety of tumor types, but its function in LUAD is uncertain.

**Methods:**

Bioinformatics analysis was conducted to evaluate the clinical value, functional enrichment, genetic correlation, and immune infiltration of *FLT3LG* in LUAD. We then used a mouse model to detect immune cell infiltration and relevant protein expression by flow cytometry and immunohistochemistry under anti-PD-1 treatment after overexpression of *FLT3LG*. The serum FLT3LG expression in LUAD patients was detected via ELISA, and PD-L1 expression in tumor samples was detected by immunohistochemistry.

**Results:**

In LUAD patients, a better prognosis is associated with elevated *FLT3LG* expression. Among the genes strongly associated with *FLT3LG*, the majority were involved in immune-related processes and were enriched predominantly in immune-related pathways. Moreover, high expression of *FLT3LG* was significantly positively correlated with increased infiltration of multiple immune cells, including T cells and natural killer (NK) cells, in lung adenocarcinomas, as well as the expression of several immune cell markers, such as CD4 and CD8a. In a mouse model, overexpression of FLT3LG in mice subjected to subcutaneous graft tumor elicited a pronounced immune response and could enhance the efficacy of anti-PD-1 therapy.

**Conclusion:**

FLT3LG could be considered as a diagnostic and prognostic marker for LUAD and might play a role in enhancing the therapeutic response to immunotherapy in patients with LUAD.

**Supplementary Information:**

The online version contains supplementary material available at 10.1186/s12885-025-14220-x.

## Background

Lung cancer has the highest incidence of malignant tumors and the highest fatality rate globally, and it is a significant threat to human health. Approximately 85% of lung cancer cases are non-small cell lung cancer (NSCLC), and lung adenocarcinoma (LUAD) is the most common subtype of lung cancer worldwide [1]. Conventional treatment modalities, including surgery, radiotherapy, and chemotherapy, demonstrate limited effectiveness and are associated with increased adverse effects. However, the emergence of immunotherapy, particularly immune checkpoint inhibitors (ICIs), has led to promising prospects for individuals with NSCLC, especially LUAD, in recent years.

Immune checkpoints encompass a category of immunosuppressive molecules that govern the immune response to tumors by modulating the extent of immune activation, and their aberrant expression and functionality are intricately linked to tumor development. Programmed cell death protein 1 (PD-1) is a significant immunosuppressive molecule expressed by immune cells, and the inhibition of PD-1 and associated pathways can significantly augment the antitumor efficacy of T cells [2, 3]. Multiple clinical trials have indicated the efficacy of PD-1/PD-L1-targeted monoclonal antibodies, including nivolumab (checkmate159, NEOSTAR) [4, 5], atezolizumab (LCMC3) [6], sintilimab (17013726), and pembrolizumab (keynote001, keynote010) [7], in increasing the overall survival and prognosis of individuals diagnosed with NSCLC [8]. Nevertheless, it is crucial to acknowledge that the observed therapeutic benefits vary among patients in real-world clinical applications. Presently, the primary indicators employed for assessing the responsiveness of patients to immunotherapy primarily include the expression of PD-1/PD-L1 and the tumor mutational burden (TMB); however, the predictive capacity of these indicators is limited and requires further investigation.

Fms-like tyrosine kinase receptor 3 (FLT3), alternatively referred to as fetal liver kinase 2, belongs to the class III tyrosine kinase receptor category. FLT3LG is the ligand of FLT3, a hematopoietic growth factor that exists in two primary forms: membrane-bound and soluble. It is expressed predominantly in various immune-related cells, including T cells and NK cells [9–11]. FLT3LG can enhance both the overall immune and humoral immune functions of T cells [12], promote immune reconstitution [13], and modify the immune status of the organism [14]. Notably, *FLT3LG* has been shown in clinical trials to play a role in the activation of CD4^+^ T cells and CD8^+^ T cells in hematological tumors. Furthermore, a significant correlation between *FLT3LG* and the prognosis of AML patients has been reported [15–18]. In solid tumors such as malignant melanoma, colorectal cancer and breast cancer, FLT3LG can increase immune cell activity and elicit antitumor responses through the use of diverse modalities, such as plasmids, adenoviruses, vaccines, and adjuvants [19–23]. However, the specific function and potential mechanism of *FLT3LG* in lung cancer immunotherapy have yet to be investigated.

In this study, we explored the significance of *FLT3LG* in NSCLC, particularly in LUAD for diagnostic and prognostic purposes, as well as its potential involvement in tumor development, utilizing databases such as TCGA and TIMER. Specifically, we focused on examining the correlation of *FLT3LG* with immune cell infiltration and further explored the impact of *FLT3LG* overexpression on the efficacy of anti-PD-1 immunotherapy in LUAD by establishing a subcutaneous tumor model of LLC cells in mice. These findings indicated that the upregulation of *FLT3LG* had a notable suppressive effect on tumor progression in LUAD patients. Furthermore, this upregulation exhibited a positive association with the degree of infiltration of various immune cells, patient prognosis, and the efficacy of anti-PD-1 immunotherapy. These results indicate that FLT3LG might augment the effectiveness of immunotherapy in patients with LUAD and could serve as a predictive indicator of the responsiveness of patients with LUAD to immunotherapeutic interventions.

## Methods

### Differential mRNA expression of *FLT3LG*

We obtained all types of cancer data from the Cancer Genome Atlas (TCGA) official website (https://portal.gdc.cancer.gov) and extracted the corresponding pairs of adjacent and tumor samples to analyze the differential mRNA expression of *FLT3LG* in paired samples. We used the Tumor Immune Estimation Resource 2.0 (TIMER2.0) database (http://timer.cistrome.org/) to explore the differential expression of *FLT3LG* between tumor tissues and adjacent normal tissues across all TCGA tumors. A total of 598 lung adenocarcinoma (LUAD) samples, 551 lung squamous cell carcinoma (LUSC) samples, and 108 corresponding normal tissue samples from the TCGA were used for analyses of *FLT3LG* in non-small cell lung cancer (NSCLC) patients. Additionally, normal lung tissue data from the GTEx website (https://www.gtexportal.org/home/) were obtained for unpaired analyses. These data were subsequently converted to transcripts per kilobase million (TPM) values. Repeated samples were excluded. The clinical data of these patients were downloaded at the same time. Clinical characteristics that were unavailable or unknown were considered missing values.

### Correlation analysis of *FLT3LG* expression and clinical factors

We analyzed the correlations between *FLT3LG* expression and clinical characteristics, including tumor (T) stage, node (N) stage, metastasis (M) stage, pathologic stage, location, sex, age, primary therapy outcome, residual tumor status, and smoking status, in the TCGA-LUAD and TCGA-LUSC cohorts. Normal and repeated samples were excluded. The *FLT3LG* expression levels were divided according to the median *FLT3LG* expression level. Then, we used the TCGA data mentioned above to analyze the expression of *FLT3LG* in patients with different pathological stages and T stages of NSCLC. K‒M plotter (http://www.kmplot.com/analysis/) was used to analyze the overall survival (OS) and first progression (FP) curves of patients with high or low expression of *FLT3LG*. The TCGA and GTEx data of NSCLC patients mentioned above were used for receiver operating characteristic (ROC) curve analysis via the “pROC” (1.18.0) package to evaluate *FLT3LG* in the diagnosis of NSCLC, and the corresponding curves were generated with the “ggplot2” (3.3.6) package.

### Differential expression analysis and enrichment analysis

The *FLT3LG* expression level data were extracted from the TCGA-LUAD dataset and categorized into high- and low-expression groups on the basis of the median *FLT3LG* expression. Normal and repeated samples were excluded. The “DESeq2” (1.36.0) package was used to perform differential expression analysis on the original Counts matrix of the data. All the statistically significant DEGs defined by an *adjusted p value* < 0.05 are displayed in volcano plots generated with the “ggplot2” (3.3.6) package, and genes with a|logFC| >1.5 were considered to be significantly different. To enhance the reliability of the enrichment analysis, DEGs with a logFC > 0.58 were used for subsequent enrichment analysis. Gene Ontology (GO) and Kyoto Encyclopedia of Genes and Genomes (KEGG) analyses of molecules with potential roles in *FLT3LG* were performed via the “clusterProfiler” (4.4.4) package.

### Correlation analysis and enrichment analysis

Correlation analysis between *FLT3LG* and other genes in LUAD was performed using TCGA-LUAD data, and the Spearman correlation coefficient was calculated. Normal and repeated samples were excluded. The total molecule expression data were extracted, and the correlations between *FLT3LG* and all the other molecules were analyzed in pairs. The correlated genes are partly shown in heatmaps generated with the “ggplot2” (3.3.6) package. According to the Spearman correlation coefficient, the top 5000 genes most positively associated with *FLT3LG* were selected for the next enrichment analysis to predict the function of *FLT3LG*.

### Gene set enrichment analysis

The gene set enrichment analysis (GSEA) used the genes obtained from the analysis of differential expression, and these genes were ranked according to the value of the fold change. We subsequently performed GSEA via the Molecular Signatures Database (MSigDB) Collection of the R package “clusterProfiler” (4.4.4) to explore the potential biological functions of FLT3LG. A *normalized P-adjusted value* < 0.05 and an FDR (*q-value*) < 0.25 were considered to indicate statistical significance.

### Protein network construction

The STRING database (https://cn.string-db.org/) was utilized to construct a protein‒protein interaction network that correlated with FLT3LG. The GeneMANIA database (https://genemania.org/) can be used to identify functionally identical genes from genomic and proteomic data. The GeneMANIA database (https://genemania.org/) was used to identify functionally identical genes of *FLT3LG* from genomic and proteomic data.

### Immune infiltration analysis

We utilized the Tumor Immune Estimation Resource (TIMER) database (https://cistrome.shinyapps.io/timer/) to assess the association between the *FLT3LG* expression level and the infiltration level of immune cells in tumors. Single-sample gene set enrichment analysis (ssGSEA) was performed with the package “GSVA” (1.46.0). Additionally, we assessed the correlation between the expression of *FLT3LG* and that of several immune cell markers. All these results were visualized via the package “ggplot2” (3.3.6).

### Clinical samples

We collected blood samples and paired clinical information, including the expression of PD-L1 in tumor tissues from 37 LUAD patients at the Department of Thoracic Surgery of the First Affiliated Hospital of Xi’an Jiaotong University, from May 2023 to December 2023. A total of 3–5 mL of patient blood was drawn, left to stand at room temperature for 2 h, and then centrifuged at 3000 rpm for 10 min at 4 °C, followed by aspiration of the upper serum layer and storage at -80 °C for subsequent experiments. The expression of PD-L1 in patients’ tumor tissues was assessed via immunohistochemistry (monoclonal, E1L3N, AmoyDx, China) in the Department of Pathology of the First Affiliated Hospital of Xi’an Jiaotong University. The methods of immunohistochemistry are described in detail in Sect. 2.14. Tumor cell Proportion Score (TPS) ≥ 1% were regarded as positive for PD-L1 expression. Informed consent was obtained from the patients for sample collection, and approval was obtained from the Ethics Committee of the First Affiliated Hospital of Xi’an Jiaotong University.

### ELISA

We analyzed the levels of FLT3LG in the serum samples collected above using the Human Flt-3 L ELISA Kit (Jianglai Biotech, Shanghai, China) according to the instructions. After the standards were prepared and diluted, 100 µL samples and standards were added to an ELISA plate that had been coated with antibodies in each well, and the plate was covered with a sealing membrane and incubated at 37 °C for 1 h. Then, the mixture was discarded, 100 µL of biotinylated antibody working solution was added directly to each well, the plate membrane was covered, and the mixture was incubated at 37 °C for 1 h. The plate was subsequently washed 3 times, 100 µL of enzyme conjugate working solution was added to each well, and the mixture was incubated at 37 °C for 30 min. The plate was washed 5 times, and 90 µL of substrate (TMB) was added to each well and incubated at 37 °C for 15 min in the dark. Then, termination solution was added after the plate was removed, and the OD value of each well was immediately measured at 450 nm via an enzyme counter. The concentration of FLT3LG in the samples was calculated after the standard curve was plotted.

### Cell culture and transfection

Murine Lewis (LLC, LL2) lung cancer cells were purchased from Wuhan Pricella Biotechnology Co., Ltd. (Wuhan, China) and subjected to STR cell line verification. The cells were cultivated in DMEM (BI, USA) supplemented with 10% fetal bovine serum (FBS) (Gibco, USA) at 37 °C in a 5% CO2 chamber. The recombinant adenoviral vector used to overexpress FLT3LG in mice, pLV3-CMV-*Flt3l* (murine sequence), was purchased from the MiaoLing Plasmid Platform (https://www.miaolingbio.com/), as well as the control empty adenoviral vector encoding only GFP. LL2 cells were transfected as described above with lentivirus and continuously selected with puromycin until a stable cell line was established; these cells were termed LLC-EV and LLC-FLT3LG.

### Mouse model

Five-week-old female C57BL/6 mice were purchased from the Laboratory Animal Center of Xi’an Jiaotong University. Each mouse was bred under specific-pathogen-free (SPF) conditions. All animal experiments were approved by the Ethics Committee of the First Affiliated Hospital of Xi’an Jiaotong University. LLC-EV and LLC-FLT3LG cells were resuspended in PBS at a density of 1 × 10^6^ cells/100 µl, and 100 µl of cells was injected subcutaneously into the axilla of each six-week-old mouse. The mice with transplanted tumors were divided into four groups: empty vector (LLC-EV, *n* = 6), overexpressed FLT3LG (LLC-FLT3LG, *n* = 6), anti-PD-1 treatment (LLC-EV + αPD-1, *n* = 6), and overexpressed FLT3LG with anti-PD-1 treatment (LLC-FLT3LG + αPD-1, *n* = 6). Two hundred micrograms of anti-mouse PD-1 antibody (clone RMP1-14; Leinco Technologies, Inc., USA) in 100 µl of PBS or 100 µl of PBS was injected intraperitoneally on the day after tumor transplantation, and the mice were treated every three days for a total of five times. The activity, spirits, and diets of the mice were observed every day during the experiment. The volume of the subcutaneous tumors was measured regularly and calculated as (length×width^2^)/2, and the tumor growth curve was plotted. After a period of 28 days, the mice were sacrificed by cervical dislocation after deep anesthesia. Subcutaneous tumors from the mice were removed and fixed in paraformaldehyde for immunohistochemical analysis, the spleens were extracted, and the lymphocytes were isolated for flow cytometry analysis.

### Western blotting

Cells were lysed by the addition of RIPA buffer, protease inhibitors and phosphatase inhibitors, after which the proteins were extracted. The proteins were electrophoresed via a 12% SDS‒PAGE gel (Solarbio BioTech, Inc., China) and transferred to a PVDF membrane (Millipore, Germany). Then, the membrane was blocked in 5% BSA (Solarbio BioTech, Inc., China) for 1 h, followed by incubation with primary antibodies overnight at 4 °C. After three washes with TBST buffer, the membranes were incubated with the secondary antibody at room temperature for 2 h, after which the proteins were visualized via luminescence. The following antibodies were used: anti-mouse Flt3L antibody (Polyclonal, Abbexa, UK), anti-alpha tubulin antibody (Polyclonal, Proteintech, China), and HRP-conjugated goat anti-rabbit IgG secondary antibody (SAB, USA).

### Flow cytometry

For flow cytometry, cells were isolated from the spleens of the mice. The spleens were removed from the mice after sacrifice, cut into small pieces, ground on a 70 μm filter and filtered with PBS, after which the cell suspensions were collected in centrifuge tubes. The cell suspension was centrifuged at 1500 rpm for 5 min, the pellets were resuspended in red blood cell (RBC) lysis buffer (Solarbio BioTech, Inc., China), the lysed erythrocytes were centrifuged, and the precipitate was washed twice with PBS to obtain a single-cell suspension. Lymphocytes were separated from single-cell suspensions using a mouse spleen lymphocyte isolation kit (Solarbio BioTech, Inc., China) and stained with surface antibodies according to the grouping. The following antibodies were used: FITC-conjugated anti-mouse CD3 (monoclonal, 17A2), PerCP-conjugated anti-mouse CD4 (monoclonal, GK 1.5), brilliant violet-conjugated anti-mouse CD8a (monoclonal, 53 − 6.7), APC-conjugated anti-mouse pDCA-1 (monoclonal, 927), PE-Cy7-conjugated anti-mouse CD11c (monoclonal, N418), APC-conjugated anti-mouse MHC-II (monoclonal, M5/114.15.2), PE-conjugated anti-mouse CD49b (monoclonal, DX5), APC-conjugated anti-mouse CD20 (monoclonal, SA271G2), and Brilliant Violet-conjugated anti-mouse FOXP3 (monoclonal, MF-14). All the antibodies used were purchased from Biolegend (USA). A total of 10^6^ cells in 100 µl per tube were incubated with FcR block (anti-mouse CD16/32, monoclonal, S17011E, Biolegend, USA) at 4 °C for 10 min and stained with the appropriate antibody at 4 °C in the dark for 20 min. After washing twice with PBS, flow cytometry was performed on a NovoCyte Flow Cytometer (Agilent, USA). We selected individual and live lymphocytes under the setting of FSC and SSC in at least 20,000 ungated events, further excluded doublet cells under the setting of FSC-H and FSC-A, and then sorted various types of lymphocytes according to the following gating strategies: CD3^+^CD4^+^ cells for CD4^+^ T cells, CD3^+^CD8^+^ cells for CD8^+^ T cells, CD4^+^CD25^+^FOXP3^+^ cells for Treg cells, CD20^+^ cells for B cells, CD11c^+^pDCA-1^+^ cells for pDCs, CD11c^+^MHC-II^+^ cells for cDCs, and CD49b^+^ cells for NK cells. Each group had its respective control sample. Analysis of the flow cytometry data was performed via NovoExpress (1.5.6).

### Immunohistochemistry

Patient tumor tissue samples were collected as previously described in Sect. 2.8. Mouse tumor tissue samples were obtained from the mouse model described above. Fixed samples were paraffin-embedded and sectioned, and the sections were deparaffinized and hydrated with dimethylbenzene and ethanol. The endogenous peroxidase activity of the sections was inactivated by incubation with 3% H_2_O_2_ for 15 min. After the nonspecific antigen was blocked with BSA (Solarbio BioTech, Inc., China) for 20 min, the diluted primary antibody was added dropwise, and the mixture was incubated at 4 °C overnight. The following antibodies were used: anti-human PD-L1 antibody (monoclonal, E1L3N, AmoyDx, China), anti-mouse CD8β antibody (monoclonal, EPR22331-54, Abcam, UK), anti-mouse CD4 antibody (monoclonal, 11A1, ImmunoWay, USA), anti-mouse CD20 antibody (monoclonal, 2F4, ImmunoWay, USA), anti-mouse CD11c antibody (polyclonal, ImmunoWay, USA), anti-mouse CD49b antibody (monoclonal, SN0752, SAB, USA), anti-mouse PD-1 antibody (polyclonal, ImmunoWay, USA), and anti-mouse Flt3L antibody (polyclonal, Abbexa, UK). The sections were subsequently incubated with secondary biotinylated anti-mouse/rabbit IgG (Servicebio, China) for 30 min at room temperature, after which the sections were visualized with a DAB kit (polyclonal, Servicebio, China). Finally, the nuclei were stained with hematoxylin solution for 5 min. All the images were obtained under a bright field microscope (Nikon, Japan). All the results were analyzed by ImageJ (1.52a).

### Statistical analysis

Statistical data analyses were performed using R software (version 4.2.1.) and GraphPad Prism (9.5.0). Paired samples of continuous variables that conformed to a normal distribution were analyzed via paired sample t tests; paired samples that did not satisfy a normal distribution were analyzed via the Wilcoxon signed rank test; and unpaired samples were analyzed via the Mann‒Whitney U test. Comparisons of categorical variables were made via the chi-square test or the Yates correction test. For comparisons of multiple samples, samples that met a normal distribution and had uniform variance were tested by one-way ANOVA and Tukey’s HSD test; for samples that met the normal distribution but did not have uniform variance, the Welch one-way ANOVA test and Games-Howell test were used; for samples that did not meet the normal distribution and did not have uniform variance, the Kruskal‒Wallis test and Dunn’s test were used. Spearman analysis was used to examine the associations among quantitative variables. All the statistical tests were two-sided, and the level of significance was set at a *P value* < 0.05.

## Results

### Expression of *FLT3LG* in pan-cancer and NSCLC

**Fig. 1:**
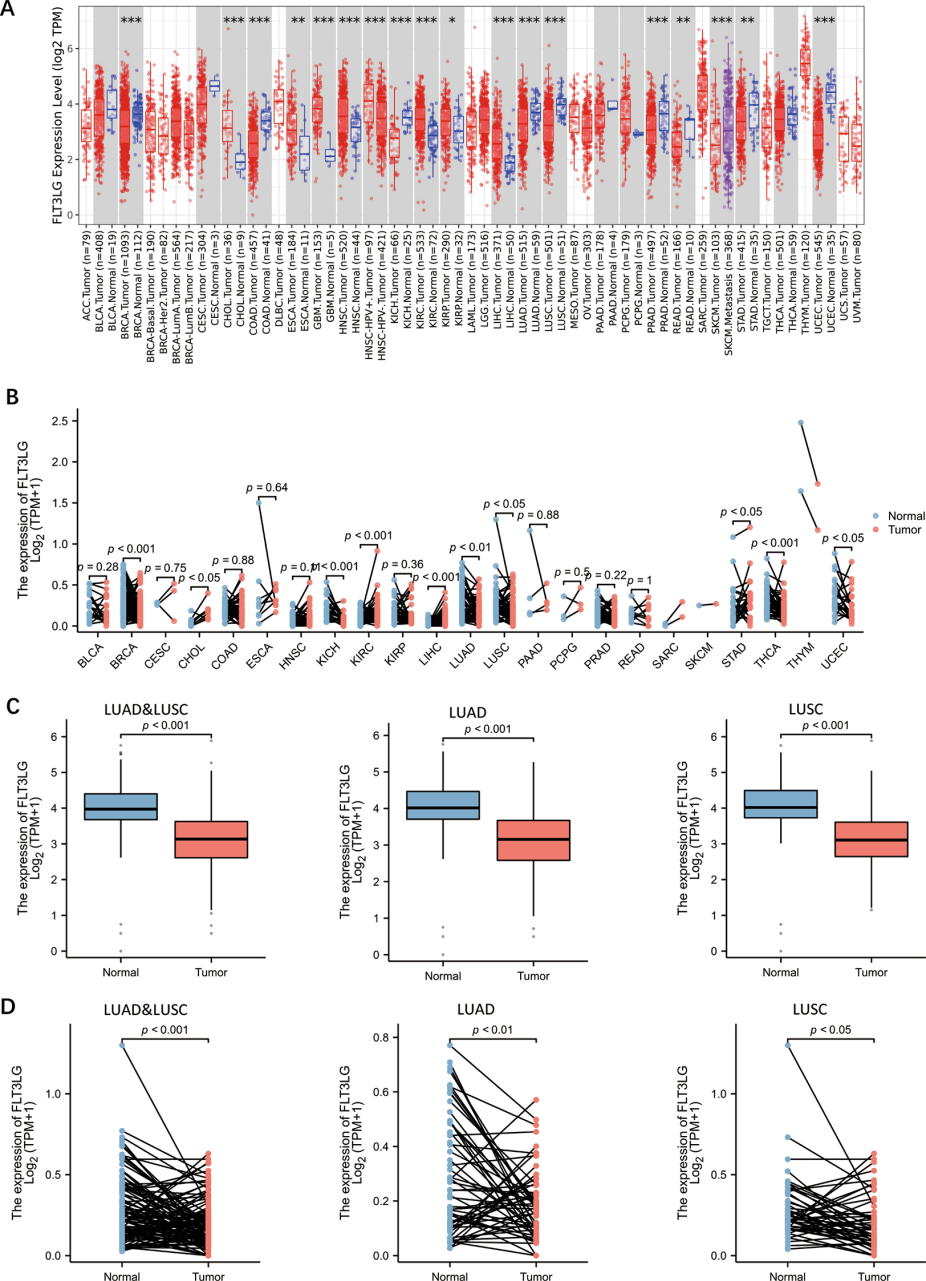
The expression of *FLT3LG* across cancers and in NSCLC. **a**. Expression of *FLT3LG* in tumor and normal tissues in the TCGA pan-cancer dataset. (*: *p* value < 0.05; **: *p* value < 0.01; ***: *p* value < 0.001) **b**. *FLT3LG* expression in tumor and paired normal tissues in the TCGA and GTEx pan-cancer datasets. **c**. *FLT3LG* expression in tumor and normal tissues from patients with NSCLC. **d**. *FLT3LG* expression in tumor and paired normal tissues from patients with NSCLC

To investigate the function of *FLT3LG* in different cancers, we first performed a pan-cancer analysis of *FLT3LG* in 8112 tumor and 1520 normal samples from the TCGA database. In total, forty types or subtypes of tumors had *FLT3LG* expression data. Compared with that in normal tissues, *FLT3LG* expression is significantly downregulated in 13 types or subtypes of tumors, including lung adenocarcinoma (LUAD) and lung squamous cell carcinoma (LUSC). In contrast, five tumor types presented higher FLT3LG expression than normal tissues did (Fig. 1a). In addition, we used paired samples from the TCGA database to analyze 7 types of tumors, including LUAD and LUSC, whose expression was significantly downregulated (Fig. 1b). Next, we compared the differential expression of *FLT3LG* between NSCLC tissues and normal lung tissues. Among the 1013 unpaired tumor samples and 397 normal tissue samples from the TCGA and GTEx combined data, we found that *FLT3LG* was expressed at significantly lower levels in both LUAD and LUSC tissues than in normal lung tissues; these results were similar to those of the analysis of 58 paired LUAD and 49 paired LUSC samples (Fig. 1c and d).

### Clinical correlation analysis and prognostic value of *FLT3LG* in NSCLC

**Fig. 2:**
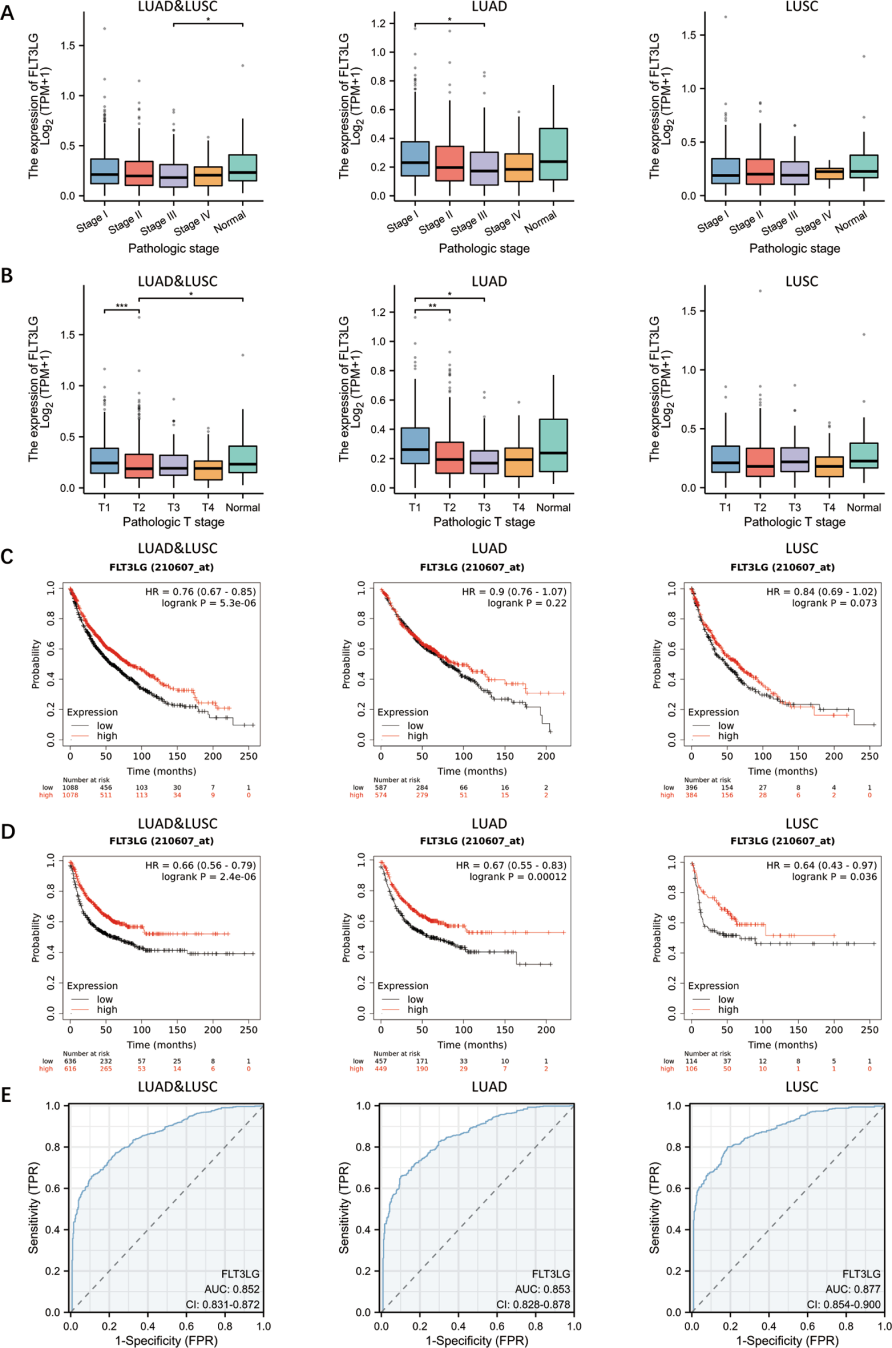
Role of *FLT3LG* in the diagnosis and prognosis prediction of NSCLC. **a**. The expression of *FLT3LG* in different pathological stages of NSCLC. **b**. The expression of *FLT3LG* in patients with different T stages of NSCLC. **c**. K‒M analysis of OS in LUAD, LUSC, LUAD and LUSC. **d**. K‒M analysis of first progression (FP) in LUAD and LUSC tissues and LUAD and LUSC tissues. **e**. The predictive value of *FLT3LG* in patients with NSCLC. (LUAD and LUSC, AUC = 0.852, CI = 0.831–0.872; LUAD, AUC = 0.853, CI = 0.828–0.878; LUSC, AUC = 0.877, CI = 0.854–0.900). *: *p* < 0.05; **: *p* < 0.01; ***: *p* < 0.001

Since a significant decrease in *FLT3LG* expression was observed in the tumor tissues of NSCLC patients, we investigated the potential associations between *FLT3LG* expression and the clinicopathological characteristics of NSCLC patients. To accomplish this, we utilized the TCGA-LUAD and TCGA-LUSC datasets to analyze the correlation between *FLT3LG* expression and various patient characteristics. Our analysis included a total of 1017 NSCLC samples, consisting of 516 LUAD samples and 501 LUSC samples. Notably, we observed significant associations between *FLT3LG* expression and T stage, sex, primary therapy outcome and smoking status. In the initial stage of tumor development (T1), a greater percentage of patients exhibited higher expression of *FLT3LG*, whereas in the subsequent stages (T2, T3 and T4) was opposite. Males and smokers exhibit lower expression of *FLT3LG*. Following primary therapy, patients classified as having progressive disease (PD) or a partial response (PR) had a greater incidence of low *FLT3LG* expression. Among the 516 LUAD samples, there was a significant correlation between *FLT3LG* expression and T stage, N stage, primary therapy outcome, and smoking status. This correlation trend was consistent with the aforementioned results. However, in LUSC, no significant correlation was observed between the *FLT3LG* expression level and any clinical characteristic (Table 1). To further investigate the diagnostic value of FLT3LG in NSCLC, we analyzed *FLT3LG* expression in patients with different T stages and pathological stages. The results revealed significant differences in *FLT3LG* expression between patients with different pathological stages (stage III vs. normal, *P* < 0.05) and patients with different pathological T stages (T1 vs. T2, *P* < 0.001; and T2 vs. normal, *P* < 0.05) in NSCLC, including both LUAD and LUSC. Additionally, in LUAD patients, *FLT3LG* expression was significantly related to pathological stage (stage I vs. stage III, *P* < 0.05) and pathological T stage (T1 vs. T2, *P* < 0.01; T1 vs. T3, *P* < 0.05). However, there was no significant difference in *FLT3LG* expression in these stages of LUSC (Fig. 2a and b). Furthermore, Kaplan‒Meier survival curves revealed that high expression of *FLT3LG* in NSCLC patients was associated with improved overall survival (OS) and first progression (FP) (Fig. 2c and d). In addition, receiver operating characteristic (ROC) analysis revealed that the expression level of *FLT3LG* has high diagnostic value for NSCLC (LUAD and LUSC: AUC: 0.852; LUAD: AUC: 0.853; and LUSC: AUC: 0.877) (Fig. 2e). These findings indicate that FLT3LG may serve as a potential molecular marker for identifying NSCLC.

**Table 1:** Associations between FLT3LG and clinicopathological features

Characteristics	LUAD&LUSC			LUAD			LUSC		
	Low expression of FLT3LG	High expression of FLT3LG	*P* value	Low expression of FLT3LG	High expression of FLT3LG	*P* value	Low expression of FLT3LG	High expression of FLT3LG	*P* value
n	508	509		258	258		250	251	
T stage, n (%)			**0.001**			**0.006**			0.119
T1	114 (11.2%)	169 (16.7%)		67 (13.1%)	102 (19.9%)		48 (9.6%)	66 (13.2%)	
T2	309 (30.5%)	262 (25.8%)		151 (29.4%)	127 (24.8%)		156 (31.1%)	137 (27.3%)	
T3	61 (6%)	57 (5.6%)		29 (5.7%)	18 (3.5%)		32 (6.4%)	39 (7.8%)	
T4	24 (2.4%)	18 (1.8%)		11 (2.1%)	8 (1.6%)		14 (2.8%)	9 (1.8%)	
N stage, n (%)			**0.097**			**0.007**			0.246
N0	314 (31.4%)	337 (33.7%)		153 (30.4%)	179 (35.5%)		166 (33.5%)	153 (30.9%)	
N1-N3	187 (18.7%)	161 (16.1%)		101 (20%)	71 (14.1%)		82 (16.6%)	94 (19.0%)	
M stage, n (%)			0.814			0.711			0.970
M0	395 (50%)	363 (45.9%)		181 (48.7%)	166 (44.6%)		209 (50%)	202 (48.3%)	
M1	16 (2%)	16 (2%)		14 (3.8%)	11 (3%)		3 (0.7%)	4 (1%)	
Pathologic stage, n (%)			0.173			0.098			0.903
Stage I	244 (24.3%)	276 (27.5%)		124 (24.4%)	152 (29.9%)		123 (24.7%)	121 (24.3%)	
Stage II	145 (14.4%)	139 (13.8%)		64 (12.6%)	58 (11.4%)		78 (15.7%)	84 (16.9%)	
Stage III	95 (9.5%)	73 (7.3%)		50 (9.8%)	34 (6.7%)		44 (8.9%)	40 (8%)	
Stage IV	16 (1.6%)	17 (1.7%)		14 (2.8%)	12 (2.4%)		3 (0.6%)	4 (0.8%)	
Location, n (%)			0.914			0.795			0.735
Central Lung	108 (25.1%)	102 (23.7%)		34 (17.9%)	29 (15.3%)		71 (29.6%)	76 (31.7%)	
Peripheral Lung	112 (26%)	108 (25.1%)		66 (34.7%)	61 (32.1%)		47 (19.6%)	46 (19.2%)	
Gender, n (%)			**0.011**			0.158			0.161
Female	184 (18.1%)	224 (22%)		131 (25.4%)	147 (28.5%)		58 (11.6%)	72 (14.4%)	
Male	324 (31.9%)	285 (28%)		127 (24.6%)	111 (21.5%)		192 (38.3%)	179 (35.7%)	
Age, n (%)			0.199			0.229			0.459
<= 65	223 (22.5%)	206 (20.8%)		125 (25.2%)	114 (22.9%)		99 (20.1%)	91 (18.5%)	
> 65	268 (27.1%)	292 (29.5%)		121 (24.3%)	137 (27.6%)		147 (29.9%)	155 (31.5%)	
Primary therapy outcome, n (%)			**0.047**			**0.029**			0.851
PD^1^	59 (7.5%)	40 (5.1%)		43 (10%)	25 (5.8%)		17 (4.7%)	14 (3.9%)	
SD^2^	23 (2.9%)	31 (3.9%)		15 (3.5%)	22 (5.1%)		8 (2.2%)	9 (2.5%)	
PR^3^	7 (0.9%)	4 (0.5%)		4 (0.9%)	2 (0.5%)		3 (0.8%)	2 (0.6%)	
CR^4^	288 (36.5%)	337 (42.7%)		143 (33.4%)	174 (40.7%)		148 (41%)	160 (44.3%)	
Residual tumor, n (%)			0.083			0.426			0.125
R0	367 (47.3%)	376 (48.5%)		171 (47.2%)	174 (48.1%)		194 (46.9%)	204 (49.3%)	
R1	14 (1.8%)	11 (1.4%)		8 (2.2%)	5 (1.4%)		6 (1.4%)	6 (1.4%)	
R2	7 (0.9%)	1 (0.1%)		3 (0.8%)	1 (0.3%)		4 (1%)	0 (0%)	
Smoker, n (%)			**0.021**			**0.04**			0.315
No	36 (3.6%)	57 (5.8%)		29 (5.8%)	46 (9.2%)		7 (1.4%)	11 (2.2%)	
Yes	460 (46.4%)	438 (44.2%)		220 (43.8%)	207 (41.2%)		240 (49.1%)	231 (47.2%)	

^1^PD: Progressive disease

^2^SD: Stable disease

^3^PR: Partial response

^4^CR: Complete response

### Functional enrichment and analysis of DEGs of *FLT3LG* in LUAD

**Fig. 3:**
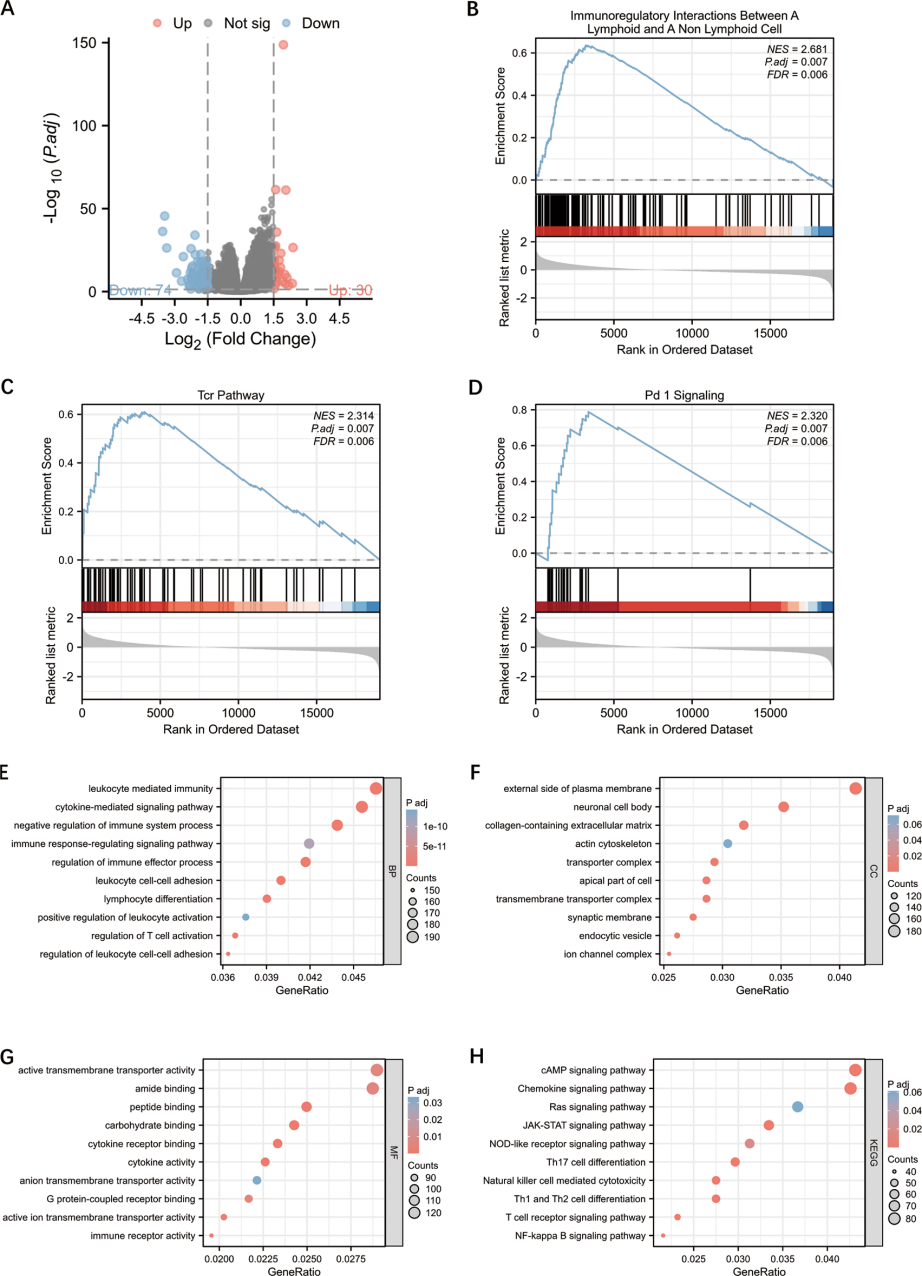
Differential gene analysis and differential gene enrichment analysis of *FLT3LG* in LUAD. **a**. Volcano plot of all differentially expressed genes (DEGs). **b**-**d**. Gene set enrichment analysis (GSEA) of *FLT3LG* based on DEGs. **e**-**g**. Gene Ontology (GO) analysis of the upregulated DEGs. (**e**. Biological process analysis; **f**. Cellular component analysis; **g**. Molecular function analysis). **h**. KEGG pathway analysis of upregulated DEGs

To further explore the potential function of FLT3LG, we initially conducted a differential expression analysis of *FLT3LG* in NSCLC. Given the lack of significant disparities in LUSC observed in previous clinical correlation analyses, our subsequent investigations focused primarily on LUAD. We utilized TCGA-LUAD data to compare 258 samples with high *FLT3LG* expression with 258 samples with low *FLT3LG* expression as the control group. Through this analysis, we discovered a total of 104 genes that were differentially expressed. Among these genes, 30 genes were upregulated, whereas 74 genes were downregulated in LUAD tumor samples compared with normal samples (adjusted *P value < 0.05*,|log2-fold change|>1.5) (Fig. 3a). The results of the DEG analysis were subsequently subjected to gene set enrichment analysis (GSEA). The results obtained from GSEA indicated that a majority of the signaling pathways that exhibited significant enrichment, as determined by their normalized enrichment score (NES), were related to the immune system (Fig. 3b and d). Owing to the limited number of DEGs identified using a threshold of|log2-fold change|>1.5, we opted to utilize DEGs defined by|log2-fold change|>0.58 for subsequent Gene Ontology (GO) and KEGG analyses to increase the effectiveness of the enrichment analysis. A significant proportion of these DEGs were specifically linked to immunologic functions and pathways (Fig. 3e and h).

### Gene correlation and enrichment analyses of *FLT3LG* in LUAD

**Fig. 4:**
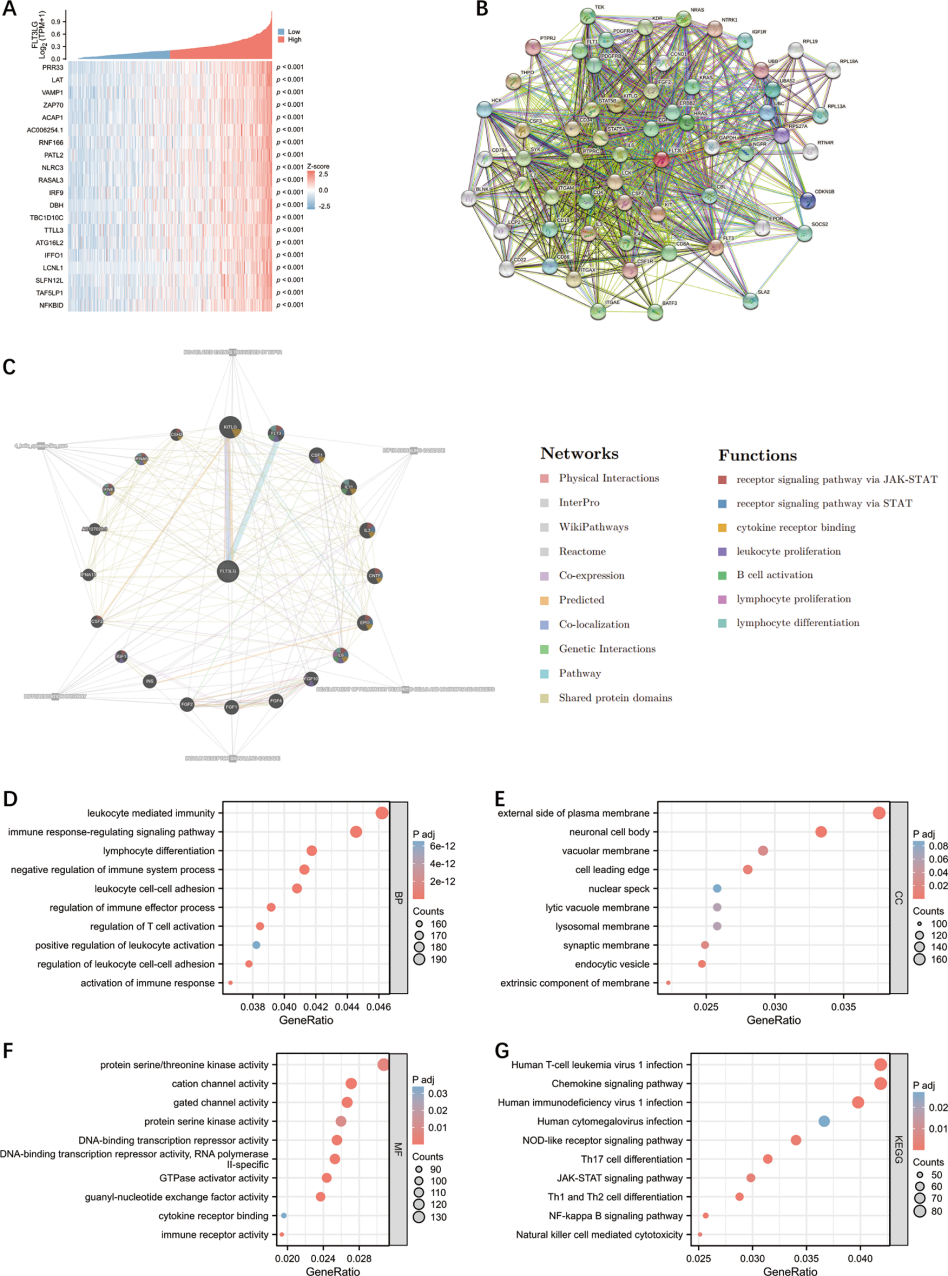
Correlated gene analysis of *FLT3LG* in LUAD. **a**. Heatmap of the top 20 genes correlated with *FLT3LG*. **b**. PPI networks constructed with STRING. **c**. PPI networks constructed in GeneMANIA. **d**-**f**. Gene Ontology (GO) analysis of the top 5000 correlated genes. (**d**. Biological process analysis; **e**. Cellular component analysis; **f**. Molecular function analysis) **g**. KEGG pathway analysis of the top 5000 correlated genes

To gain a deeper understanding of the potential functions of FLT3LG and its potential associated pathways, we conducted a correlation analysis between *FLT3LG* and other genes in LUAD. A heatmap was constructed and shows the top 20 genes that presented the strongest positive correlation with *FLT3LG* (Fig. 4a). We subsequently performed GO and KEGG analyses of the top 5000 genes. GO analyses of the related genes revealed that *FLT3LG* was associated primarily with immune-related gene terms (Fig. 4d and f). Like the enrichment analysis of DEGs mentioned earlier, KEGG analysis of co-expressed genes revealed a close association between *FLT3LG* and immune pathways, particularly those related to T cells, natural killer (NK) cells and virus infection (Fig. 4g). Additionally, we used the STRING database (cn.string-db.org) and GeneMANIA (genemania.org) to independently investigate the potential interacting proteins and genes of *FLT3LG* (Fig. 4b and c). These functions clearly exhibit a brief association with the immune system. Collectively, these findings suggest that in LUAD, FLT3LG expression primarily correlates with immune-related functions and pathways, potentially influencing immune cell activities.

### The correlation between *FLT3LG* level and the degree of immune cell infiltration in LUAD patients

**Fig. 5:**
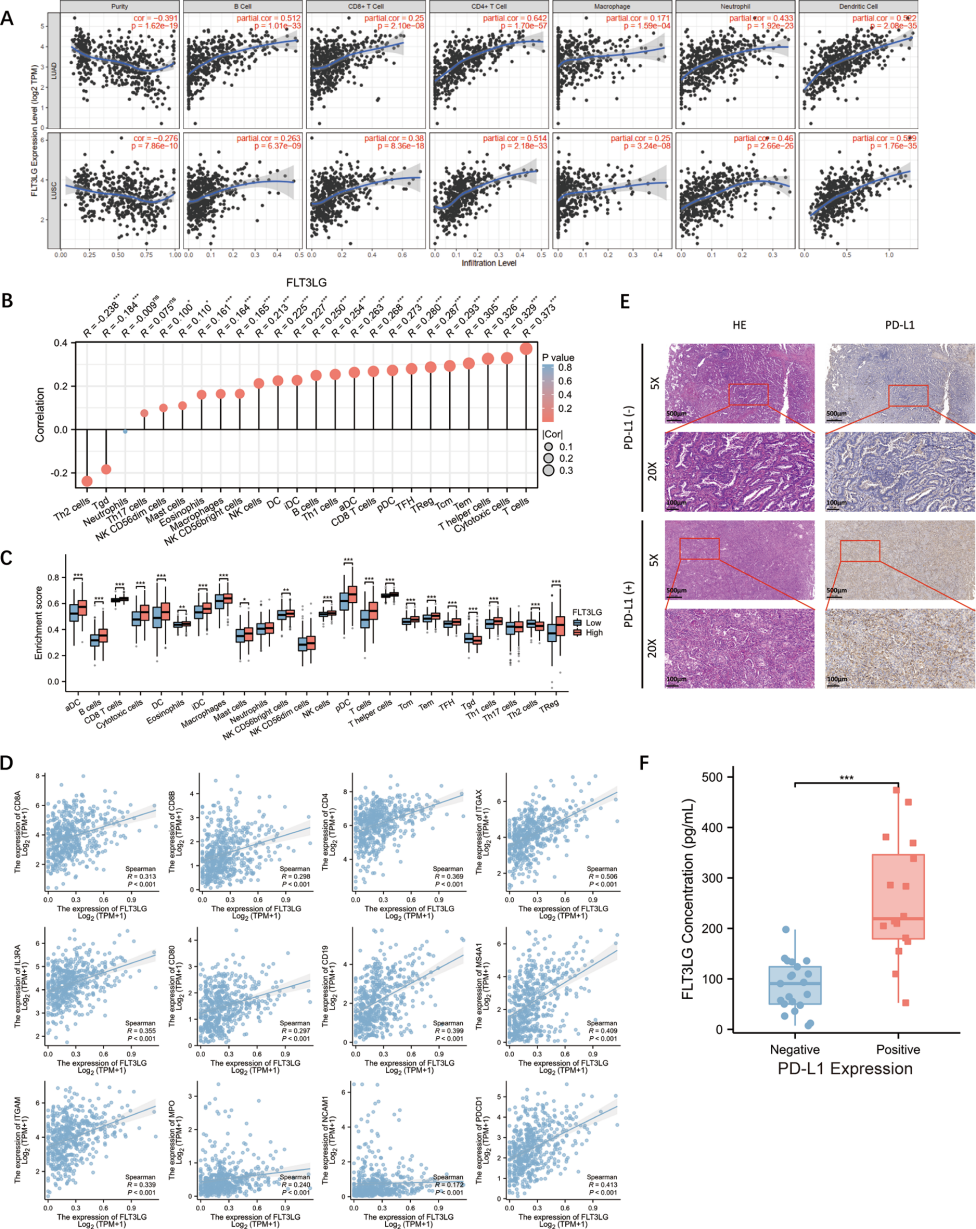
Correlations between *FLT3LG* expression level and immune cell infiltration and the expression of immune signature. **a**. Correlation between the expression of *FLT3LG* and the infiltration of different immune cells according to TIMER. **b**. Correlation between the expression of *FLT3LG* and the infiltration of different immune cells in the TCGA cohort. **c**. Immune cell infiltration levels in patients with different expression levels of *FLT3LG*. **d**. Correlation between *FLT3LG* expression and immune cell marker expression. **e**. HE staining and PD-L1 immunohistochemical staining of tumor samples from LUAD patients. **f**. Serum FLT3LG concentrations in LUAD patients with negative or positive PD-L1 expression in tumor tissue samples. *: *p* < 0.05; **: *p* < 0.01; ***: *p* < 0.001

Since enrichment analysis revealed a significant association between FLT3LG and tumor immunity in LUAD, we subsequently proceeded to examine the correlation between the *FLT3LG* expression level (TPM) and the extent of immune cell infiltration in the TIMER database and TCGA-LUAD dataset quantified by ssGSEA. The results revealed a positive correlation between the *FLT3LG* expression level and the infiltration of various common immune cell types, such as B cells, CD8^+^ T cells, CD4^+^ T cells, natural killer (NK) cells, macrophages and dendritic cells (DCs), with a particular emphasis on different subtypes of T cells (Fig. 5a and c). We further analyzed the correlation between *FLT3LG* expression and various immune signatures, including *CD8A*, *CD8B*, and *CD4* for T cells; *CD11c (ITGAX)*, *CD123 (IL3RA)*, and *CD80* for DCs; *CD19* and *CD20 (MS4A1)* for B cells; *CD11b (ITGAM)* for granulocytes; *MPO* for macrophages; *CD56 (NCAM1)* for NK cells; and *PD-1 (PDCD1)*. Our findings revealed a significant correlation between the *FLT3LG* expression level and the expression levels of these markers, particularly *CD11c*, *CD20*, and *PD-1* (Fig. 5d). These findings suggest that FLT3LG may be associated primarily with the infiltration of DCs, T cells, and B cells in the tumor microenvironment in lung cancer patients. Since tumor *PD-L1* expression significantly influences the response of patients to immunotherapy, we conducted an initial verification of the correlation between serum FLT3LG expression and PD-L1 expression in clinical tumor tissue samples. The clinical information of the patients is listed in Supplemental Table 1. We divided LUAD patients into two groups by PD-L1 expression via immunohistochemistry (Fig. 5e) and measured the level of FLT3LG in the serum of these patients via ELISA. We found that the serum FLT3LG level in the PD-L1-positive group was significantly higher than that in the PD-L1-negative group (*P* < 0.001, Fig. 5f). In addition, when we divided serum FLT3LG expression into high and low groups according to the median FLT3LG level, we detected a significant positive correlation between serum FLT3LG expression and PD-L1 expression in tumor samples from LUAD patients (*P* < 0.001, Table 2). These data substantiate the findings of the correlation and enrichment analysis, indicating that FLT3LG potentially contributes to immune-related functions in LUAD.

**Table 2:** Correlation of serum FLT3LG and PD-L1 expression in LUAD tissues

		FLT3LG		r	*P* value
		Low (*n* = 18)	High (*n* = 19)		
PD-L1	Negative (*n* = 21)	16	5	0.631	< 0.001
	Positive (*n* = 16)	2	14		

### FLT3LG can promote the effect of anti-PD-1 therapy in LUAD mouse models

**Fig. 6:**
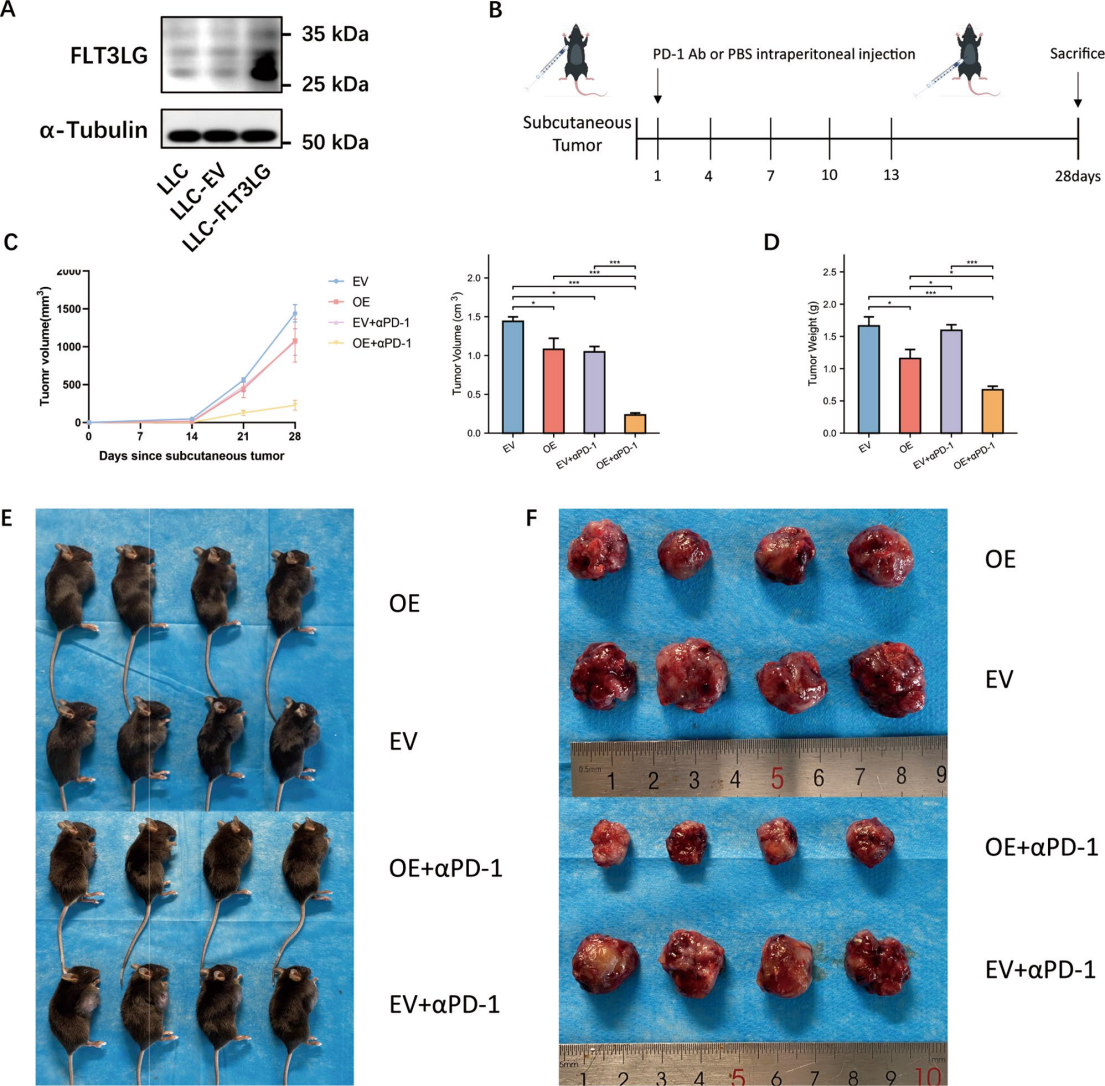
Effect of FLT3LG on anti-PD-1 therapy efficacy in a mouse LUAD model. **a**. Transfection of LLC cells. The blots were cut prior to hybridization with antibodies during blotting. **b**. Flowchart of the in vivo experiments. **c**. Growth curves and volume of LLC subcutaneous tumors. **d**. Weights of the LLC subcutaneous tumors. **e**. Mice bearing LLC subcutaneous tumors. **f**. LLC subcutaneous tumors. *: *p* < 0.05, **: *p* < 0.01, ***: *p* < 0.001

To evaluate the immune function of FLT3LG in vivo, we established a mouse subcutaneous graft tumor model. Since we aimed to investigate the tumor immune microenvironment, we needed to use C57BL/6 mice, which have normal immune systems, so we used the mouse LUAD cell line Lewis Lung Carcinoma (LLC) to establish a subcutaneous tumor model; therefore, the sequence of Flt3L in the recombinant adenoviral vector was a murine sequence. LLC-EVs were transfected with empty vectors, while LLC-FLT3LG cells were transfected with an *Flt3L*-overexpressing lentivirus (Fig. 6a). A total of 24 six-week-old C57BL/6 mice were divided into four groups: empty vector (EV, *n* = 6), overexpressed FLT3LG (OE, *n* = 6), anti-PD-1 treatment (EV + αPD-1, *n* = 6), and overexpressed FLT3LG with anti-PD-1 treatment (OE + αPD-1, *n* = 6). After the subcutaneous injection of the transfected cells into the axilla of the mice, the mice were administered intraperitoneal injections of 200 µg of anti-PD-1 antibody or PBS every three days for a total of five injections (Fig. 6b). All the mice were sacrificed after 28 days. We observed mouse survival, measured the tumor volume periodically, and weighed the tumors after sacrifice. Significant differences in tumor volume and tumor weight were observed between the OE group and the EV group. Notably, the combination of OE and anti-PD-1 had the most obvious inhibitory effect on the tumor growth rate, tumor volume and tumor weight (Fig. 6c and d). These findings collectively suggest that FLT3LG not only inhibits the growth and proliferation of LUAD tumors to a certain degree but also enhances the therapeutic efficacy of anti-PD-1 therapy in LUAD (Fig. 6e and f).

### Enhancement of anti-PD-1 therapeutic effect by FLT3LG might be associated with the promotion of immune cells proliferation and infiltration

**Fig. 7:**
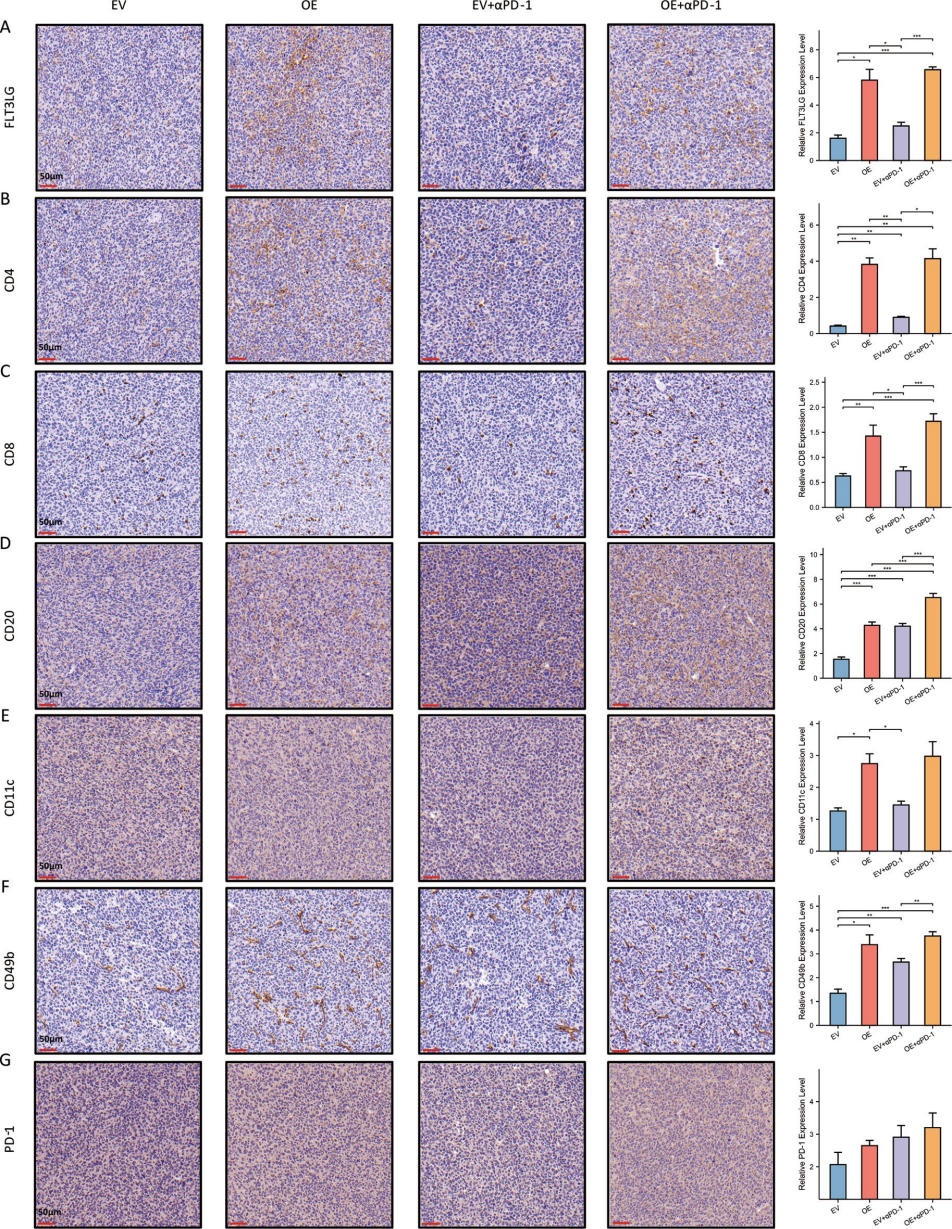
Overexpression of FLT3LG can promote the infiltration of diverse immune cells in the TME. Subcutaneously transplanted tumors from mice were excised for immunohistochemical staining to analyze the expression of multiple immune-related molecules. **a**. The expression of FLT3LG in the four groups. b-f. The expression of several common immune cell markers. (**b**. CD4^+^ T cells; **c**. CD8^+^ T cells; **d**. CD20^+^ B cells; **e**. CD11c^+^ DCs; **f**. CD49b^+^ NK cells.) **g**. PD-1 expression in the four groups. Scale bar, 50 μm. *: *p* < 0.05, **: *p* < 0.01, ***: *p* < 0.001

**Fig. 8:**
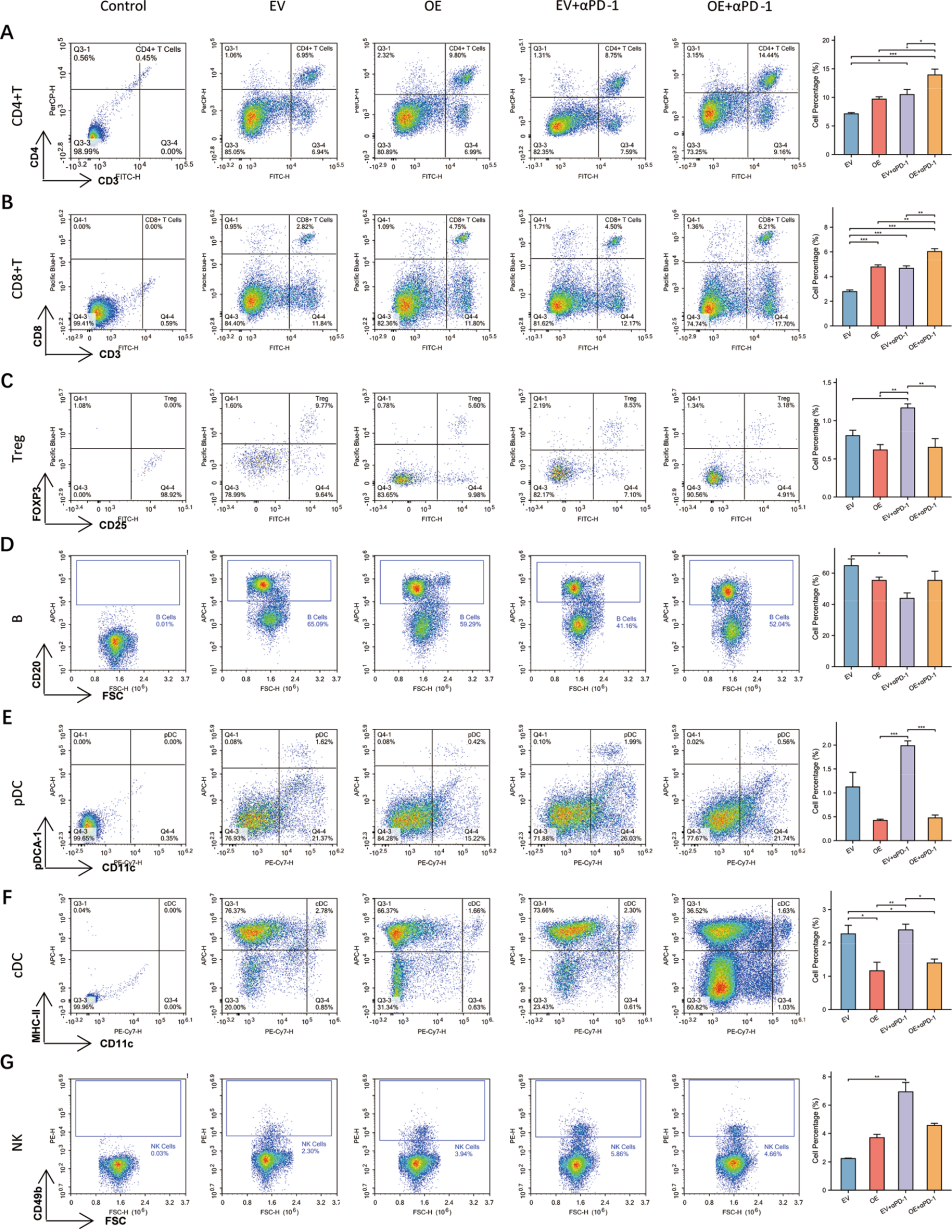
Flow cytometry analysis of immune cells from the spleen of a LUAD subcutaneous tumor mouse model. Lymphocytes isolated from the spleens of mice with subcutaneous LUAD tumors were evaluated via flow cytometry. **a**. CD4^+^ T cells (CD3^+^CD4^+^). **b**. CD8^+^ T cells (CD3^+^CD8^+^). **c**. Treg cells (CD4^+^CD25^+^FOXP3^+^). **d**. B cells (CD20^+^). **e**. pDC (CD11c^+^pDCA1^+^). **f**. cDC (CD11c^+^MHCII^+^). **g**. NK cells (CD49b^+^). *: *p* < 0.05, **: *p* < 0.01, ***: *p* < 0.001

Since we found that FLT3LG can enhance the effect of anti-PD-1 therapy in LUAD, we wanted to further investigate the underlying mechanisms responsible for this effect. After the aforementioned mice were sacrificed, we performed immunohistochemistry (IHC) analysis of the tumor samples. Previous research has revealed strong correlations between FLT3LG and T cells, B cells, DCs, and NK cells; therefore, we focused on these several cell types in subsequent analyses. Increased expression of FLT3LG significantly enhanced the infiltration of multiple immune cells, particularly T cells and NK cells, into the tumor microenvironment (Fig. 7a and f). However, the impact on PD-1 expression was not pronounced (Fig. 7g). Furthermore, the spleens were extracted and subjected to lymphocyte isolation for subsequent flow cytometric analysis to assess the overall immunization status (Fig. S1). Compared with those of the other three groups, OE + anti-PD-1 treatment significantly increased the proportions of CD4^+^ T cells and CD8^+^ T cells in the spleen. Furthermore, compared with EV treatment alone, OE treatment significantly increased the number of CD8^+^ T cells in the spleen (Fig. 8a and b). As Treg cells play an important role in autoimmunity and were mentioned in our previous results, we conducted an analysis of these specific types of T cells. Our results indicated that Treg cells were significantly lower in the OE + anti-PD-1 group than in the EV + anti-PD-1 group. However, no statistically significant differences were observed between the OE and EV groups (Fig. 8c). Additionally, compared with EV + anti-PD-1 treatment, OE + anti-PD-1 treatment led to a noticeable reduction in the pDC and cDC populations in the spleen (Fig. 8e and f). However, there was no significant effect on B cells (Fig. 8d) or NK cells (Fig. 8g). On the basis of the present data, the potential of FLT3LG to augment the effectiveness of anti-PD-1 therapy may be attributed to the promotion of immune cell proliferation and infiltration in TME (tumor microenvironment), with a particular emphasis on T cells.

## Discussion

In this study, we employed bioinformatics analysis of data from The Cancer Genome Atlas (TCGA) and other databases, as well as in vivo experiments in animals, to investigate the association and impact of FLT3LG on the diagnosis and treatment of NSCLC, particularly in relation to immune infiltration of tumors and the efficacy of immunotherapy. The findings revealed that, compared with that in normal lung tissues, *FLT3LG* expression in tumor tissues was significantly lower in NSCLC patients. Moreover, a positive correlation between the expression level of *FLT3LG* and the prognosis of patients was observed, suggesting its potential as a biomarker for prognostic assessment in NSCLC. Furthermore, *FLT3LG* is closely associated with the functions of diverse immune cell types and is significantly positively correlated with the extent of immune cell infiltration, especially in lung adenocarcinoma (LUAD). Our study successfully demonstrated that the overexpression of FLT3LG in a C57BL/6 mouse model of lung cancer effectively inhibited tumor growth, increased the infiltration of immune cells in the TME, enhanced the activity of various immune cells within the spleen, and improved the therapeutic efficacy of the PD-1 antibody.

FLT3LG functions as a hematopoietic growth factor and primarily operates during the developmental phase of diverse immune cells. It facilitates the expansion, growth, activation, and maturation of dendritic cells (DCs) while also synergistically promoting the generation of early T cells and natural killer (NK) precursor cells in conjunction with other hematopoietic factors [9, 12, 14, 24]. Therefore, prior investigations have predominantly concentrated on examining its stimulatory impact on immune cell proliferation. Hence, initial investigations concerning FLT3LG in the clinical domain have similarly prioritized hematological malignancies or autoimmune disorders. These studies have demonstrated a noteworthy correlation between FLT3LG and the incidence and therapeutic management of acute myeloid leukemia, rheumatoid arthritis, systemic lupus erythematosus, allergic asthma, and various other ailments [17, 25–29]. In recent years, reports have indicated that FLT3LG can facilitate the proliferation of immune cells, impede tumor growth either directly or indirectly, and augment the effectiveness of anti-PD-1 therapy in solid tumors [30–33]. However, the literature rarely addresses the importance of FLT3LG in the prognostic evaluation and diagnosis of tumors. Our investigation commenced with the examination of differential expression between cancerous and healthy tissues, revealing a noteworthy decrease in *FLT3LG* expression in NSCLC compared with normal lung tissues, and the higher the expression is, the better the prognosis of patients. Furthermore, ROC curve analysis revealed that *FLT3LG* had an area greater than 0.85 in both the LUAD and LUSC subtypes of NSCLC. These findings suggest that *FLT3LG* is a valuable diagnostic and prognostic marker for NSCLC.

To further investigate the potential underlying factors contributing to the strong correlation between *FLT3LG* and the diagnosis and prognosis of LUAD, we conducted an enrichment analysis of both differentially expressed genes (DEGs) and genes associated with FLT3LG. Our findings revealed that *FLT3LG* primarily participated in tumor immune-related functions and pathways within the context of LUAD. Moreover, a subsequent analysis of immune infiltration revealed a significant positive correlation between the expression level of *FLT3LG* and the infiltration of various immune cells, including CD8^+^ T cells, CD4^+^ T cells, Treg cells, B cells, pDCs, and others. To ascertain the functional role of FLT3LG in vivo, we conducted an experiment in which *FLT3LG* was overexpressed in the mouse lung cancer cell line LLC. These cells were then utilized to establish a subcutaneous transplantation tumor model in C57BL/6 mice via concurrent administration of anti-PD-1 therapy. The findings revealed that the overexpression of FLT3LG in tumor cells effectively impeded tumor growth, augmented the proliferation of CD4^+^ and CD8^+^ T cells, suppressed the activity of Treg cells, and significantly potentiated the efficacy of anti-PD-1 therapy. These results indicate that FLT3LG may play a role in the immune response to LUAD tumors by facilitating the proliferation and infiltration of immune cells, thereby potentially influencing the efficacy of immunotherapy. Prior research has demonstrated the involvement of FLT3LG in the activation of CD4^+^ and CD8^+^ T cells, consequently impacting the prognosis of AML patients [15], and reduced levels of FLT3LG have been proposed as a diagnostic marker for cervical cancer and have been found to be correlated with the extent of immune cell infiltration within the tumor [34]. Furthermore, the potential of FLT3LG to enhance tumor immunotherapy efficacy has been demonstrated in various tumor types, such as glioma, melanoma, breast cancer, and colorectal cancer, as supported by previous studies [32, 33, 35, 36]. These findings effectively corroborate our results, but investigations of FLT3LG in relation to immunotherapy in LUAD patients are limited. Despite the availability of multiple biomarkers for diagnosis and prognosis prediction in LUAD, indicators specifically associated with immunotherapy and its effectiveness in this context are scarce. Presently, the assessment of immunotherapy responsiveness relies primarily on evaluating PD-L1 expression and the tumor mutational burden (TMB), yet the overall prognosis is unsatisfactory [37–39]. Since the expression level of FLT3LG is significantly correlated with the activity and infiltration of diverse immune cells in LUAD, along with the abundance of immune cells in the tumor microenvironment, and can, to some extent, determine the therapeutic efficacy in patients with tumors [40, 41], it is plausible that the expression level of FLT3LG may be a potential predictor of the effectiveness of immunotherapy in patients with LUAD.

In previous studies, the administration of FLT3LG involved the injection of synthetic adenoviruses, vaccines, recombinant proteins, or similar agents into the bodies or tumors of the subjects under investigation to promote overall immune cell proliferation or immune cell infiltration in the tumor microenvironment, thereby indirectly inducing antitumor effects through the modulation of immune cells and immune status [32, 33, 42, 43]. In this study, we transfected lentiviruses to construct mouse LLC cells with elevated expression of FLT3LG and subsequently used these cells to establish a subcutaneous transplantation tumor model in the axilla, a lymphoid tissue-rich region. This experimental design not only enables investigation of the impact of increased *FLT3LG* expression on tumor growth and proliferation but also facilitates analysis of the influence of secreted FLT3LG on the regulation of various immune cell activities in the spleen, which may contribute to subsequent exploration of the underlying mechanism through which FLT3LG enhances the effectiveness of anti-PD-1 therapy.

Despite obtaining a generally positive outcome in our study, certain inadequacies remain. First, our research focused primarily on bioinformatics analysis of *FLT3LG* and subsequently validated previous findings and hypotheses through in vivo experiments. However, we currently have insufficient clinical data to support the conclusions. In the preceding section, we previously highlighted that certain investigations posited that the presence of FLT3LG in the serum may serve as an indicator of comprehensive immune status and the efficacy of oxaliplatin chemotherapy. Consequently, it is plausible to hypothesize that the presence of FLT3LG in the serum could predict the outcome of immunotherapy in LUAD patients. Moreover, the detection of FLT3LG in serum offers a more expedient alternative to immunohistochemistry. Henceforth, our research endeavors will persist in the acquisition of pertinent clinical data, encompassing serum samples obtained from patients diagnosed with LUAD both prior to and subsequent to immunotherapy. These data will be subjected to meticulous measurement and analysis to enhance the comprehensiveness of our study. Moreover, when verifying the effect of FLT3LG on LUAD in vivo, because we were conducting only a preliminary exploration, we focused solely on the overexpression of *Flt3l* in tumor cells, aiming to elucidate the influence of FLT3LG on LUAD and immunotherapy under conditions of heightened expression. We subsequently plan to knock out *Flt3l in vivo* to suppress the function of the FLT3LG protein in vivo, potentially leading to further discoveries. Furthermore, our findings indicate that FLT3LG expression is significantly correlated with the proliferation of immune cells and the level of immune cell infiltration in the TME and that a high level of FLT3LG enhances the efficacy of anti-PD-1 therapy in patients with LUAD. There might be an association between these two results, but the present research findings do not yet prove that there is a direct correlation between them. This is one of the limitations and deficiencies of this study. In our subsequent investigations, we will further investigate specific mechanisms, such as the signaling pathways involved in the FLT3LG-mediated regulation of immune cell infiltration, to better understand the potential mechanisms by which FLT3LG enhances the efficacy of immunotherapy in LUAD.

## Conclusion

In conclusion, our study utilized bioinformatics analysis and animal experiments to demonstrate that, in NSCLC, the expression of *FLT3LG* was significantly lower in tumor tissues than in normal tissues, and we observed a positive correlation between the degree of *FLT3LG* expression and patient prognosis. Furthermore, in LUAD, FLT3LG expression was found to impact the penetration of immune cells in the tumor microenvironment and the overall quantity of immune cells, and these findings suggest that FLT3LG expression has the potential to increase the efficacy of anti-PD-1 immunotherapy. Hence, FLT3LG is postulated to be a diagnostic and prognostic biomarker for LUAD, and augmenting the expression of FLT3LG in LUAD patients could improve the therapeutic efficacy of anti-PD-1 therapies. Consequently, further investigations into the underlying mechanism of FLT3LG and tumor immunity may provide novel insights for improving the prognosis of LUAD patients.

## Electronic supplementary material

Below is the link to the electronic supplementary material.

Supplementary Material 1

Supplementary Material 2

Supplementary Material 3

## Data Availability

Data is available from the corresponding author upon reasonable request. The datasets supporting the conclusions of this article were available in the TCGA database and GTEx website: https://portal.gdc.cancer.gov/projects/TCGA-LUAD, https://portal.gdc.cancer.gov/projects/TCGA-LUSC, https://www.gtexportal.org/home/.

## Abbreviations

NSCLC: Non-small cell lung cancer
PD-1: Programmed cell death protein 1
FLT3LG: Fms-related tyrosine kinase 3 ligand
TME: Tumor microenvironment
GSEA: Gene set enrichment analysis
NK: Natural killer
DC: Dendritic cell
OS: Overall survival
PD-L1: Programmed cell death-Ligand 1
AML: Acute myeloid leukemia
TCGA: The Cancer Genome Atlas
LLC: Lewis lung carcinoma
LUAD: Lung adenocarcinoma
LUSC: Lung squamous cell carcinoma
FP: First progression
ROC: Receiver operating characteristic
AUC: Area under the curve
GO: Gene Ontology
KEGG: Kyoto Encyclopedia of Genes and Genomes
SPF: Specific-pathogen-free
PBS: Phosphate-buffered saline
DMEM: Dulbecco’s modified Eagle medium
SDS‒PAGE: Sodium dodecyl sulfate–polyacrylamide gel electrophoresis
PVDF: Polyvinylidene fluoride
BSA: Bovine serum albumin
HRP: Horseradish peroxidase
DAB: Diaminobenzidine
DEG: Differentially expressed gene
NES: Normalized enrichment score
BP: Biological process
CC: Cellular composition
MF: Molecular function
MHC: Major histocompatibility complex
