# Supplementary figures and images for: FLT3LG modulates the infiltration of immune cells and enhances the efficacy of anti-PD-1 therapy in lung adenocarcinoma
Source: BMC Cancer. 2025 May 6;25:831. doi: 10.1186/s12885-025-14220-x (PMC12057023; doi:10.1186/s12885-025-14220-x)

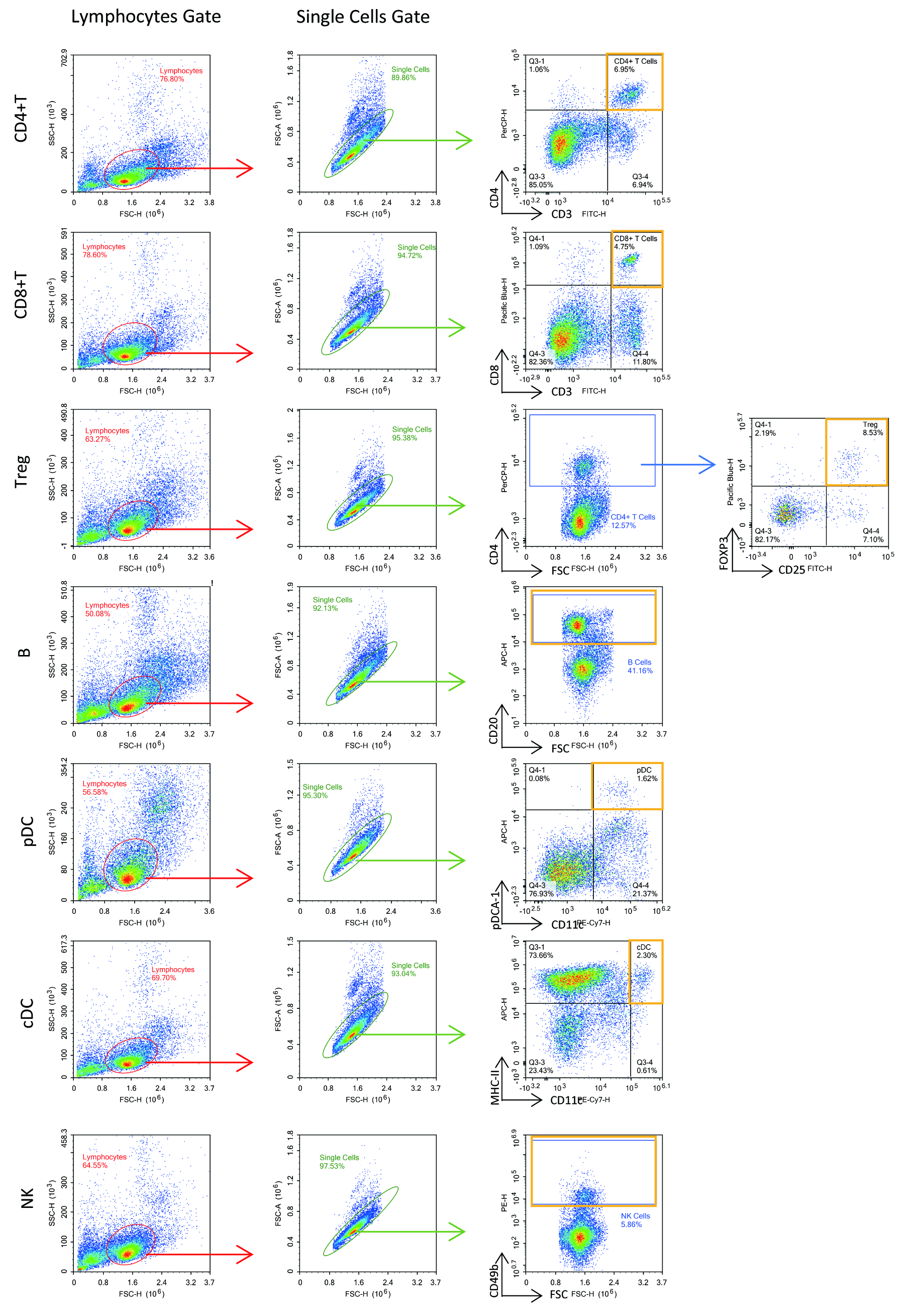

Supplement: Supplementary file 1 — Supplementary Material 1 [file 12885_2025_14220_MOESM1_ESM.tif]

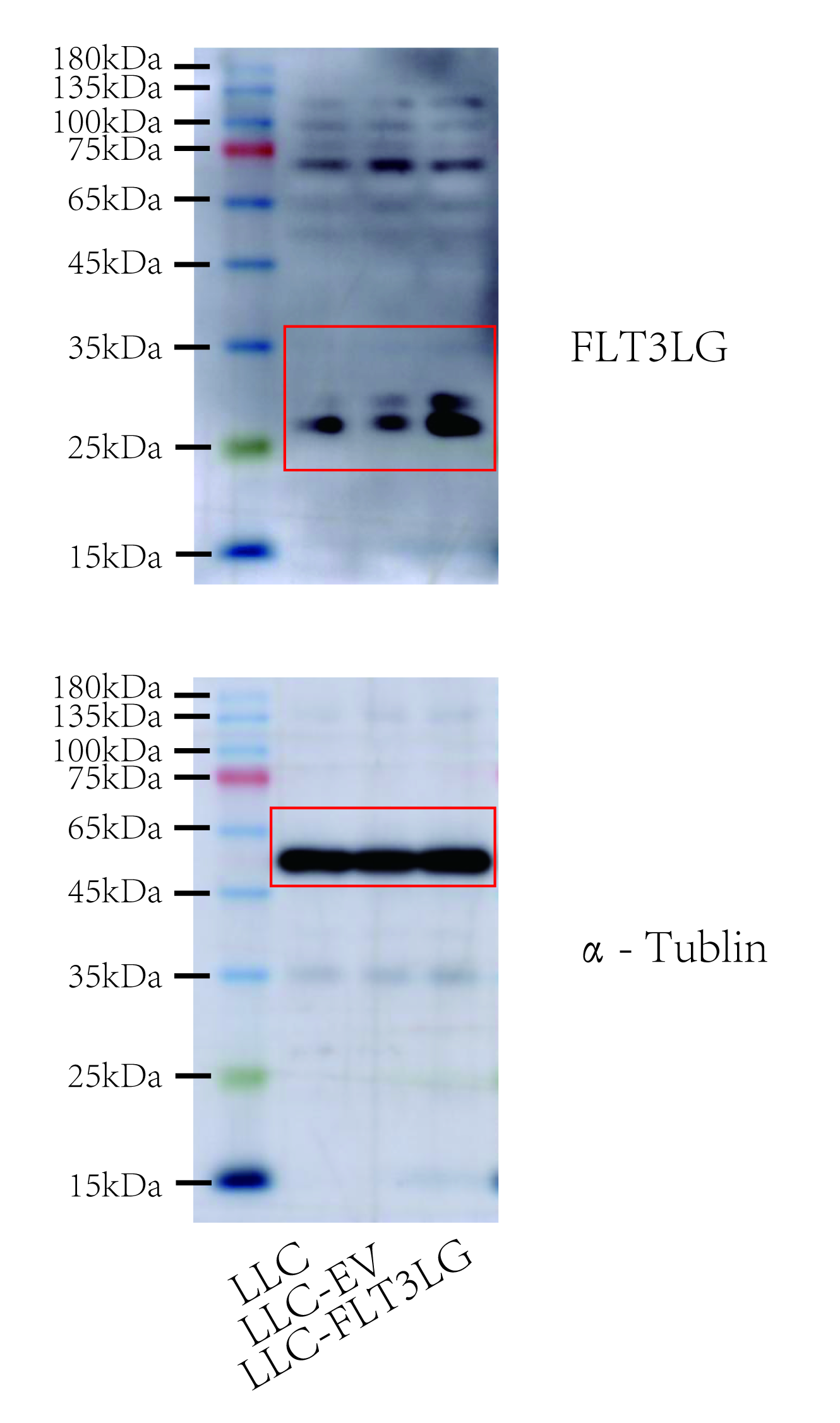

Supplement: Supplementary file 3 — Supplementary Material 3 [file 12885_2025_14220_MOESM3_ESM.tif]
